# P-113. Reduction in Non-Ventilator Hospital Acquired Pneumonias After Implementation of an Alcohol-Based Antiseptic for Nasal Decolonization

**DOI:** 10.1093/ofid/ofae631.320

**Published:** 2025-01-29

**Authors:** Louise A Zalusky-Kamm, Tyron White

**Affiliations:** Monument Health Rapid City, RAPID CITY, South Dakota; Monument Health, Rapid City, South Dakota

## Abstract

**Background:**

Hospital-acquired pneumonia (HAP), particularly non-ventilator hospital-acquired pneumonia (NV-HAP), poses significant challenges including extended hospital stays, increased readmissions, costs, and mortality, yet remains understudied. Current guidelines do not address skin and nasal decolonization (ND) for NV-HAP prevention. This study evaluates the impact of incorporating an alcohol-based antiseptic (ABA) for ND within a universal skin decolonization protocol on NV-HAP rates.

NV-HAP Reduction Pre and Post Implementation of Nasal Decolonization CY2021-2024YTD

**Figure 1:**
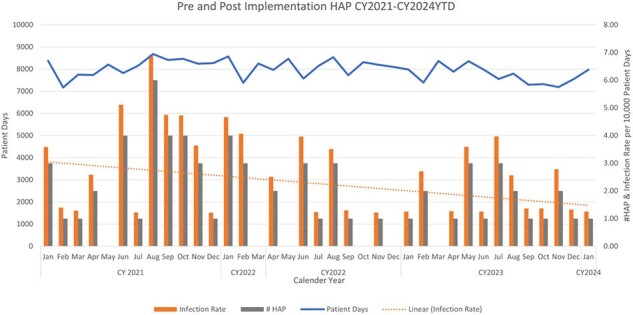
NV-HAP Reduction Pre and Post Implementation nasal Decolonization CY2021-2024YTD. Nasal Decolonization with nasal antiseptic added to established antimicrobial wipe protocol March 16th, 2021.

**Methods:**

We conducted a retrospective observational study comparing NV-HAP incidences in two cohorts: pre-intervention without ND and post-intervention with ABA ND. NV-HAP events were identified using the National Health Safety Network (NHSN) definition for non-ventilator-associated pneumonia [PNEU]. ND was applied to patients identified as high risk or in critical care. Nasal antiseptic was administered upon admission and every 12 hours until discharge. Daily universal skin decolonization continued per protocol using antiseptic wipes.

Non-Ventilator Hospital Acquired Pneumonia with S. aureus

**Figure 2:**
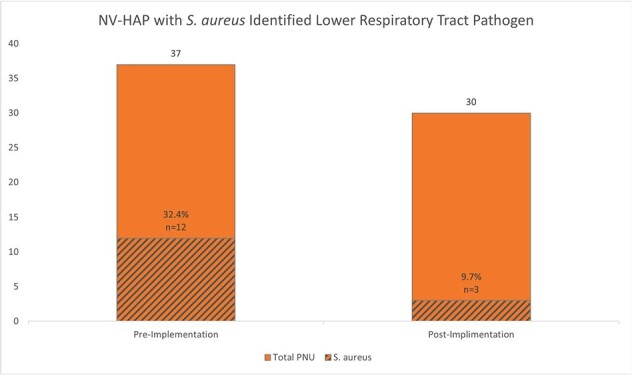
NV-HAP with S. aureus reduction represented pre and post Implementation of nasal decolonization with nasal antiseptic.

**Results:**

The pre-implementation phase (January 2021-March 2022) identified 37 NV-HAP events over 121,496 patient days (rate: 0.305 per 1,000 patient days). Post-implementation (April 2022-January 2024), we observed 30 NV-HAP events across 173,661 patient days (rate: 0.173 per 1,000 patient days), reflecting a 43% reduction rate. Statistical analysis using the NHSN rate calculator showed a significant reduction with a p-value of 0.02. Additionally, post-implementation data revealed a notable 75% decrease in *Staphylococcus aureus* cultures for NV-HAP cases [71% decrease in Methicillin-resistant S. aureus (MRSA)]. These results underscore the effectiveness of adding ABA for ND to a universal skin decolonization protocol in significantly lowering NV-HAP events and reducing prevalent pathogens like *S. aureus*.

**Conclusion:**

The addition of an ABA for ND significantly reduced NV-HAP events. Additionally, the decrease in NV-HAP was also associated with a decrease in overall *S. aureus* cultures. Adding an ABA for ND to a universal skin decolonization protocol is an effective intervention to reduce NV-HAP.

**Disclosures:**

**Louise A. Zalusky-Kamm, B.S. CLS, MT, CIC**, Global Industries- Nozin: Grant/Research Support|Global Industries- Nozin: One-time financial assistance to attend APIC annual conference and speak at Nozin symposium. **Tyron White, BS CLS, MLS(ASCP)SM , CIC**, Global Life Technologies Corp: Speaker: Lodging and 1 day conference costs covered by company at APIC 2023

